# Exogenous Glutathione Increases Arsenic Translocation Into Shoots and Alleviates Arsenic-Induced Oxidative Stress by Sustaining Ascorbate–Glutathione Homeostasis in Rice Seedlings

**DOI:** 10.3389/fpls.2019.01089

**Published:** 2019-09-13

**Authors:** Ha-il Jung, Myung-Suk Kong, Bok-Rye Lee, Tae-Hwan Kim, Mi-Jin Chae, Eun-Jin Lee, Goo-Bok Jung, Chang-Hoon Lee, Jwa-Kyung Sung, Yoo-Hak Kim

**Affiliations:** ^1^Division of Soil and Fertilizer, National Institute of Agricultural Science, Rural Development Administration, Wanju, South Korea; ^2^Department of Animal Science, Institute of Agricultural Science and Technology, College of Agriculture and Life Science, Chonnam National University, Gwangju, South Korea; ^3^Department of Fruit Science, Korean National College of Agriculture and Fisheries, Jeonju, South Korea

**Keywords:** arsenic toxicity, ascorbate–glutathione cycle, glutathione, reactive oxygen species, rice

## Abstract

Glutathione (GSH) plays diverse roles in the physiological processes, stress defense, growth, and development of plants. This study investigated the effects of exogenous GSH on the biochemical responses of reactive oxygen species and antioxidant levels in rice (*Oryza sativa* L. cv. Dasan) seedlings under arsenic (As) stress. As treatment inhibited growth; increased the level of superoxide, hydrogen peroxide, and malondialdehyde; and enhanced the uptake of As by the roots and shoots in hydroponically grown 14-day-old seedlings. Furthermore, it reduced GSH content and GSH redox ratios, which have been correlated with the decrease in ascorbate (AsA) redox state. Whereas the exogenous application of GSH in As-treated seedlings reduced As-induced oxidative stress, improved antioxidant defense systems by maintaining antioxidant and/or redox enzyme homeostasis, and increased the AsA and GSH contents, the GSH application also increased the As translocation from the roots to the shoots. These results indicated that the increase in GSH redox state can be linked to an increase in the AsA redox ratio *via* the induction of the AsA–GSH cycle. Therefore, the results suggest that exogenous GSH application should be a promising approach to enhance As stress resistance in rice plants.

## Introduction

Arsenic (As) pollution in agricultural soils and waters, which is rapidly increasing worldwide due to industrialization and urbanization, causes serious environmental issues and adversely influences crop yield and quality (Zhu et al., 2008; Khan et al., 2010; Akinbile and Haque, 2012; Kim et al., 2016). Social and industrial activities, such as mining and smelting, have resulted in aggressive expansion of As-contaminated regions, including paddy soils. Because contamination of rice by As might cause serious health risks to the end consumer in the long term, it is a matter of considerable importance (Xie and Huang, 1998; Roychowdhury et al., 2002; Williams et al., 2005; Jung et al., 2017b).

In plants, As disturbs protein functions due to its high affinity to their sulfhydryl group. Moreover, it disrupts the cell membrane through intracellular lipid peroxidation by generating reactive oxygen species (ROS), such as superoxide anion radical (O_2_^•−^), hydrogen peroxide (H_2_O_2_), and hydroxyl radicals (OH^•^), leading to apoptosis (Meharg and Hartley-Whitaker, 2002; Finnegan and Chen, 2012; Dave et al., 2013; Farooq et al., 2016). The ROS generated by As toxicity accumulates in the plant cells. This can severely deteriorate the normal biological functions or processes not only by inducing a negative effect on the maintenance of redox homeostasis but also by impairing the biosynthesis of basic substances that support plant growth, such as carbohydrates, proteins, fats, and nucleic acids (Singh et al., 2013; Petrov et al., 2015; Singh et al., 2015a). However, plants attempt to overcome As toxicity by activating antioxidant scavenging systems that can alleviate the oxidative stress caused by excessive absorption and accumulation of As (Jung et al., 2015; Singh et al., 2016; Sharma et al., 2017). The alleviation of As-induced oxidative stress and maintenance of redox homeostasis are mostly achieved by two biochemical scavenging systems. The first is an enzymatic antioxidant scavenging system based on several identified enzymes, including superoxide dismutase (SOD), catalase (CAT), peroxidase (POX), ascorbate (AsA) POX (APX), monodehydroascorbate reductase (MDHAR), dehydroascorbate (DHA) reductase (DHAR), and glutathione (GSH) reductase (GR). The second is a non-enzymatic antioxidant scavenging system, such as AsA and GSH, which is reactivated and regulated to scavenge excessive ROS accumulated in the cells. In the first step of the AsA–GSH cycle, APX catalyzes the reduction of H_2_O_2_ to H_2_O using AsA as an electron donor. As a consequence, AsA is oxidized to DHA. GSH is used as an electron donor by DHAR to reconvert DHA to AsA. The oxidized form of GSH is GSH disulfide (GSSG), which can be recycled to GSH by GR using reduced nicotinamide adenine dinucleotide phosphate (NADPH) as the electron donor. Therefore, these various components of the AsA–GSH cycle play a pivotal role in a coordinated manner and protect cells against oxidative damage caused by As toxicity (Foyer and Noctor, 2011; Noctor et al., 2012; Dave et al., 2013; Farooq et al., 2016).

The roles of antioxidant enzymes in scavenging the ROS produced by As are well identified through numerous studies in a variety of plants (Panda et al., 2010; Rahman et al., 2015; Singh et al., 2015a; Singh et al., 2015b). However, studies on both the reduction of oxidative stress caused by heavy metals and the application of GSH, which plays a central role in *in vivo* redox regulation, are limited (Mostofa et al., 2014; Kim et al., 2017; Sharma et al., 2017; Semida et al., 2018). Noctor et al. (2012) reported a comprehensive and systematic result on how GSH plays an important role in alleviating oxidative stress, by reviewing studies that examined the effect of GSH application on numerous plants subjected to various environmental abiotic stresses, such as drought, salinity, and extreme temperatures. Moreover, Chen et al. (2010) reported that hydroponic treatment with GSH reduces the generation of ROS due to Cd toxicity in barley. Mostofa et al. (2014) and Shri et al. (2009) reported the effects of GSH, as a component of the antioxidant scavenging system, on reducing Cu and As toxicity in rice seedlings, respectively. Furthermore, Semida et al. (2018) demonstrated reduced heavy metal toxicity in cucumber seedlings and increased heavy metal tolerance in plants subjected to GSH treatment. Despite several studies on GSH treatment as an antioxidant to alleviate heavy metal-induced oxidative stress, there are only a few studies on the relationship between As toxicity and GSH.

Rice (*Oryza sativa* L.) is one of the most important agricultural crops worldwide, including in South Korea, and several studies have evaluated its ability of As uptake from soil (Akinbile and Haque, 2012; Kim et al., 2016; Jung et al., 2017b). However, information on the relationship between GSH and antioxidant scavenging systems in rice plants is still unclear, especially with regard to As phytotoxicity. We therefore conducted a hydroponic experiment using rice seedlings to elucidate the effects of exogenous GSH application on As uptake, As-induced modulation of ROS and lipid peroxidation, and the antioxidant scavenging system from As exposure. The results of this study can be applied to develop a practical approach to reduce As phytotoxicity in plants, especially rice.

## Materials and Methods

### Plant Cultivation and Experimental Procedure

Rice (*O. sativa* L. cv. Dasan) seeds were sterilized with 70% ethanol for 2 min, washed extensively with distilled water, surface-sterilized by incubation in 2 ml L^−1^ of agrimycin solution (commercial bactericide, 8% ipconazole) for 48 h, and rinsed thoroughly with distilled water. The seeds were then germinated in distilled water before being incubated at 30°C for 48 h. Uniformly germinated seeds were transferred to a hydroponic growth system with hydroponic solution. Uniform two-leaf stage seedlings were transplanted into plastic pots (12 plants per pot) for cultivation. These pots (30 cm × 25 cm × 15 cm, 0.075-m^2^ surface area) were filled with 9 L of hydroponic solution containing the following macroelements and microelements: 1 mM of NH_4_NO_3_, 0.6 mM of NaH_2_PO_4_·H_2_O, 0.3 mM of K_2_SO_4_, 0.2 mM of CaCl_2_, 0.4 mM of MgCl_2_·6H_2_O, 45 µM of Fe-EDTA, 50 µM of H_3_BO_3_, 9 µM of MnCl_2_·4H_2_O, 0.3 µM of CuSO_4_·5H_2_O, 0.7 µM of ZnSO_4_·7H_2_O, and 0.1 µM of Na_2_MoO_4_·2H_2_O. The pH of the solution was adjusted to 5.6, and the hydroponic solution was replaced every 7 days (Kamachi et al., 1991). The hydroponically cultivated seedlings were grown in a greenhouse (National Institute of Agricultural Science, Wanju, Republic of Korea) with natural sunlight, day/night temperatures of 30°C/25°C, and also day/night relative humidity of 60%/80%. Four-leaf seedlings were then grown in hydroponics treated with 15 µM of As (NaAsO_2_) and were simultaneously sprayed with GSH (50 and 100 mg kg^−1^) and 2 ml L^−1^ of commercial surfactant (10% polyoxyethylene alkyl aryl ether and 20% sodium lignosulfonate). GSH was applied just once using a handheld sprayer delivering 1,000 L ha^−1^ of solution through flat-fan spray tips (Jung et al., 2018). Fourteen days after treatment, fresh 100-mg samples of roots and leaves were frozen in liquid nitrogen and stored at −80°C until ROS and antioxidant analyses.

### Plant Growth Response and Analysis

The effect of GSH application on the growth characteristics of rice seedlings grown in As-treated hydroponics with or without the antioxidant was evaluated by measuring plant height, shoot fresh weight (FW), shoot dry weight (DW), root DW, and shoot water content (WC). After the 14-day treatment period, plant height was measured, the seedlings were separated into roots and leaves, and the shoot FW was determined. For shoot WC measurement, the shoots were excised, and their FW was immediately recorded. For shoot DW measurement, the weighed fresh shoots were dried at 60°C for 72 h and then weighed again.

### As Content From Rice Tissues

Rice root and shoot samples were washed with deionized water for approximately 5 min and incubated at 80°C in an oven until they dried completely. The well-dried samples were then ground to a powder, and 200 mg of the powder was digested using a Graphite Block Acid Digestion System (ODLAB Co., Ltd., Seoul, Republic of Korea). After digestion, the solutions were cooled to ambient temperature, diluted to 100 ml with ultrapure water, and filtered using Grade No. 40 filter papers (Whatman, Buckinghamshire, UK). As content was determined *via* inductively coupled plasma–mass spectroscopy analysis (Agilent 7900; Agilent Technologies Inc., Santa Clara, CA, USA).

### Determination of Superoxide, Hydrogen Peroxide, and Malondialdehyde

The content of O_2_^•−^ was measured spectroscopically at 530 nm, according to the method described by Elstner and Heupel (1976); a standard NaNO_2_ curve was used for calculations.

The content of H_2_O_2_ was determined chromatically following the method described by Jana and Choudhuri (1982). The optical density and absorbance of the samples were measured at 410 nm, and H_2_O_2_ content was calculated using an extinction coefficient of 0.28 µM^−1^ cm^−1^.

Lipid peroxidation was evaluated *via* the level of malondialdehyde (MDA) by the thiobarbituric acid method of Buege and Aust (1978) with slight modifications. The absorbance of each sample at 532 nm was measured and corrected for nonspecific turbidity by subtracting the value from the absorbance at 600 nm. The content of MDA was calculated using an extinction coefficient of 156 mM^−1^ cm^−1^.

### Measurement of Antioxidant Enzyme Activities

Frozen root and leaf organs were pulverized in liquid nitrogen using a prechilled mortar and pestle and then resuspended in 100 mM of potassium phosphate buffer (pH 7.5) containing 2 mM of ethylenediaminetetraacetic acid (EDTA), 1% polyvinylpyrrolidone-40 (PVP-40), and 1 mM of phenylmethylsulfonyl fluoride (PMSF). The suspension was centrifuged at 15,000 × *g* for 20 min at 4°C, and the resulting supernatant was used directly as an enzyme source (Lee et al., 2013).

SOD (EC 1.15.1.1) activity was determined based on the capacity of the enzyme to inhibit the photoreduction of nitroblue tetrazolium (NBT) (Lee et al., 2009). One unit of SOD was defined as the amount of enzyme that inhibited the rate of NBT photoreduction by 50% at 560 nm.

CAT (EC 1.11.1.6) activity was assayed, according to the method of Lee et al. (2013), by monitoring the decline in absorbance at 240 nm for 1 min (coefficient, ε = 36 mM^−1^ cm^−1^) as a result of H_2_O_2_ degradation.

APX (EC 1.11.1.11) activity was assayed, using the method of Chen and Asada (1989), by monitoring the decrease in absorbance at 290 nm for 1 min as AsA (coefficient, ε = 2.8 mM^−1^ cm^−1^) was oxidized.

MDHAR (EC 1.6.5.4) activity was assayed following the method described by Hossain et al. (1984). The assay was determined by monitoring the oxidation rate of the NADPH by MDHAR at 340 nm for 1 min. The activity was calculated using an extinction coefficient of 6.2 mM^−1^ cm^−1^.

DHAR (EC 2.5.1.1.8) activity was assayed chromatically according to the method described by Nakano and Asada (1981). The assay was measured by monitoring the increase in absorbance at 265 nm for 1 min owing to the reduction of DHA into AsA using an extinction coefficient of 14 mM^−1^ cm^−1^.

GR (EC 1.6.4.2) activity was measured, using the method of Rao et al. (1996), by monitoring the decrease in absorbance at 340 nm as NADPH (coefficient, ε = 6.2 mM^−1^ cm^−1^) was oxidized.

### Evaluation of AsA/DHA and GSH/GSSG

The total AsA, reduced AsA, and oxidized DHA levels were measured according to the method of Law et al. (1983) with slight modifications. To colorimetrically measure the total AsA (reduced AsA plus oxidized DHA) and reduced AsA levels, 100 μl of the supernatant was mixed with 250 μl of 0.15 M of K_2_HPO_4_ buffer (containing 5 mM of EDTA, pH 7.4) in the presence of either 50 μl of 10 mM of dithiothreitol (for the total AsA) or 50 μl of water (for the reduced AsA). The samples were incubated for at least 10 min at 25°C before the addition of 50 μl of 0.5% *N*-ethylmaleimide. A color-developing solution containing 200 μl of 10% trichloroacetic acid, 200 μl of 44% *O*-phosphoric acid, 200 μl of 4% α,α′-dipyridyl in 70% ethanol, and 11 μl of 30% FeCl_3_ was added to the above mixtures. These were then vigorously mixed and incubated at 37°C for 60 min. The absorbance of each sample was recorded at 525 nm. The concentration of total and reduced AsA was determined using a calibration curve, which was prepared using different AsA concentrations. The DHA content was obtained by subtracting the reduced AsA concentration from the total AsA concentration.

The content of GSH, reduced GSH, and oxidized GSSG was measured using the method described by Meister and Anderson (1983) with the following modifications. The rice seedling tissues (100 mg) were ground to powder in liquid nitrogen with a prechilled mortar and pestle. The powder was mixed with 1.2 ml of 5% (w/v) 5-sulfosalicylic acid to form a paste in order to reduce the oxidation of GSH. The paste was then centrifuged at 12,000 × *g* for 10 min at 4°C, and the clear supernatant was collected and used for the total GSH assay in 96-well microtiter plates. The supernatant (40 µl) was added to a 160-µl reaction mixture containing 143 mM of Na phosphate buffer (pH 7.5), 6.3 mM of EDTA, 0.22 mM of NADPH, and 20 µl of 6 mM of 5,5-dithiobis-2-nitrobenzoic acid. The reaction was initiated by the addition of 0.5 units of GR, and changes in the absorbance were monitored at 412 nm for 1 min in a microplate reader. For GSSG measurement, 2 µl of 2-vinylpyridine was added to 100 µl of supernatant and mixed thoroughly for formation of the conjugates with GSH. The reaction was allowed to proceed for 1 h at 25°C in a chamber. The absorbance of the derivative samples was measured by the same method as described for the total GSH. The total GSH and GSSG concentrations in the samples were determined using linear regression obtained from a standard curve of GSH and GSSG.

### Data Analysis

**Figure 1:**
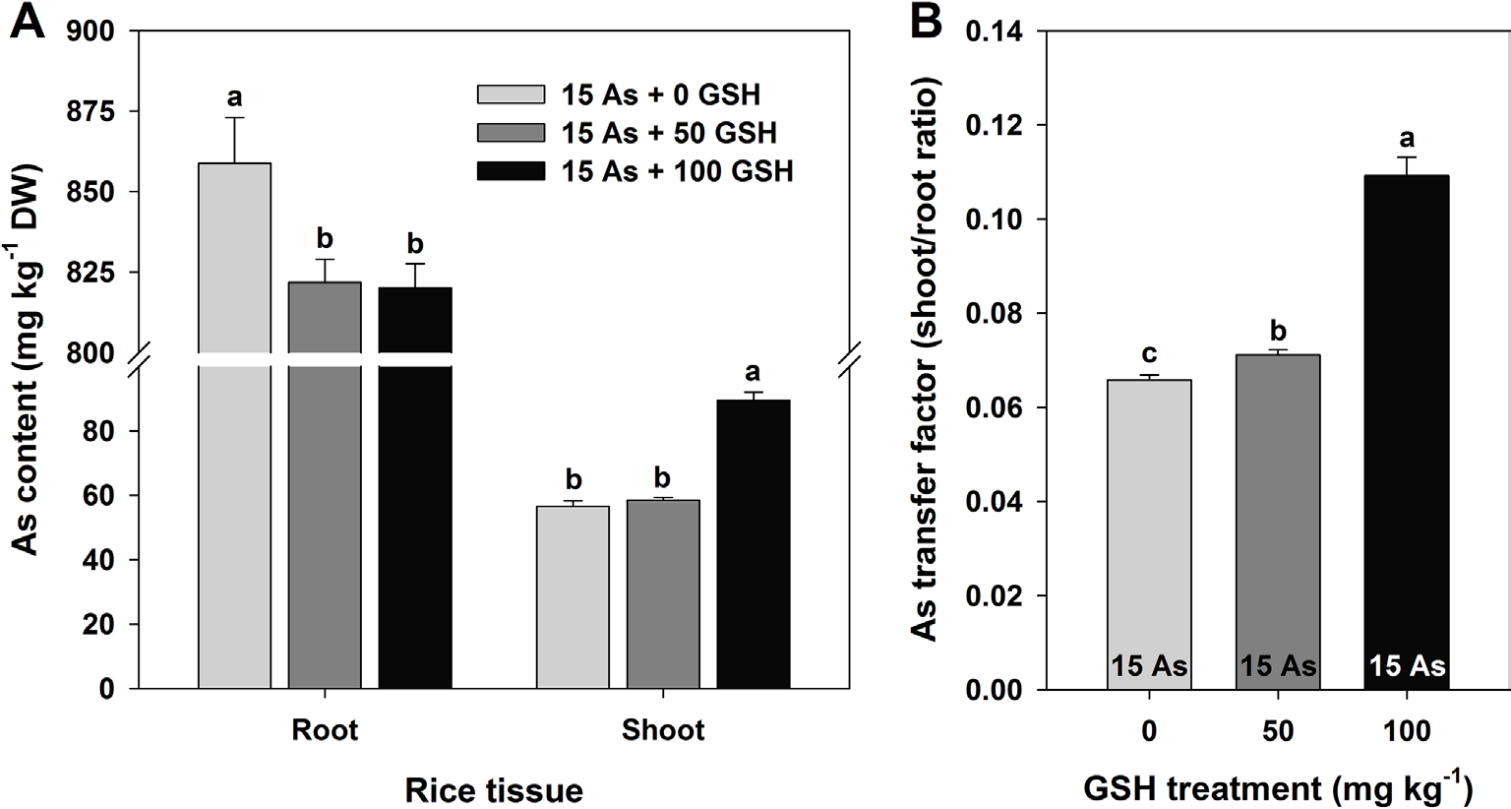
Effect of glutathione (GSH) application on the arsenic (As) content **(A)** and As ratio **(B)** between the root and shoot of rice seedlings grown in As-treated hydroponics. Rice seedlings at the four-leaf stage were cultivated in a hydroponic solution containing 15 µM of As and sprayed with GSH (50 and 100 mg kg^−1^). Fourteen days after treatment, the level of As in the roots and shoots was measured *via* inductively coupled plasma–mass spectroscopy analysis. The data are presented as mean ± standard error of the mean of three replications. Means denoted by the same letter are not significantly different at the 5% level, according to Fisher’s least significant difference (LSD) test.

Root-to-shoot As ratio was calculated as follows (**Figure 1B**): As transfer factor = *Ca/Cb*, where *Ca* is the As concentration in the shoot and *Cb* is the As concentration in the root.

To elucidate the response of targeted metabolites of oxidative stress and the AsA/GSH redox state in the roots and leaves of rice seedlings treated with different levels of GSH, correlation coefficient analysis and principal component analysis (PCA) were performed using MetaboAnalyst 3.0 (www.metaboanalyst.ca; Xia and Wishart, 2016) for heatmap generation and cluster analysis. Ward’s clustering algorithm and Pearson’s distance were applied for the cluster analysis.

All data were subjected to analysis of variance (ANOVA), and Fisher’s least significant difference (LSD) test was used to determine significant differences among treatments. The statistical analyses were carried out using a statistical analysis software (SAS ver. 9.2; SAS Institute Inc., Cary, NC, USA). Differences at *P* < 0.05 were considered significant. In the figures, the data are expressed as mean ± standard error (SE).

## Results

### Effect of GSH Application on the Growth Responses of As-Stressed Rice Seedlings

To evaluate the effect of GSH on As toxicity, the rice seedlings at the four-leaf stage were grown in 15-μM-As-treated hydroponics for 14 days, simultaneously with or without the exogenous GSH treatments (50 and 100 mg kg^−1^). Compared with the untreated plants, all As-treated seedlings exhibited As-induced growth inhibition in terms of plant height, shoot FW, and shoot DW, regardless of GSH application. However, the root DW and shoot WC were not affected by As treatment (**Table 1**). Furthermore, the seedlings treated with GSH (without As) were not significantly different on the growth responses of rice seedlings, compared with the untreated plants (data not shown).

**Table 1:** Effect of glutathione (GSH) application on the growth characteristics of rice seedlings grown in arsenic (As)-treated hydroponics. Rice seedlings at the four-leaf stage were cultivated in a hydroponic solution containing 15 µM of As and sprayed with GSH (50 and 100 mg kg^−^^1^).

Treatment	Plant height (cm)	Shoot FW (g)	Shoot DW (g)	Root DW (g)	Shoot WC (%)
Control	42a	1.85a	0.349a	0.081a	81.1a
15 µM As + 0 mg GSH kg^−1^	39b	1.42b	0.257b	0.078a	81.8a
15 µM As + 50 mg GSH kg^−1^	39b	1.51b	0.280b	0.087a	81.5a
15 µM As + 100 mg GSH kg^−1^	39b	1.46b	0.269b	0.085a	81.6a

Means within a column followed by the same letter are not significantly different at the 5% level based on Fisher’s least significant difference (LSD) tests. FW, fresh weight; DW, dry weight; WC, water content.

### Analysis of As Content and As Transfer Factor in As-Stressed Rice Seedlings

As level was significantly higher in the roots than in the shoots (stems and leaves), and the As content was 4.3–4.5% lower in both the 50 and 100 mg kg^−1^ GSH-treated seedlings than in the As-treated plants. However, there was no statistical difference detected between these two GSH treatments. Although the As content decreased in the roots in response to GSH application, the shoots showed an opposite trend in 100 mg kg^−1^ GSH treatment. The As content in the shoots was 3.4% higher in the 50 mg kg^−1^ GSH treatment and 58.3% higher in the 100 mg kg^−1^ GSH treatment, compared with that in the As-alone treatment (**Figure 1A**). The ratios of root As level to shoot As level in the 50 and 100 mg kg^−1^ GSH treatments were 1.1- and 1.7-fold higher than that in the As treatment, respectively (**Figure 1B**).

### Changes in the Production of O_2_^•−^, H_2_O_2_, and MDA in As-Stressed Rice Seedlings With or Without GSH Application

**Figure 2:**
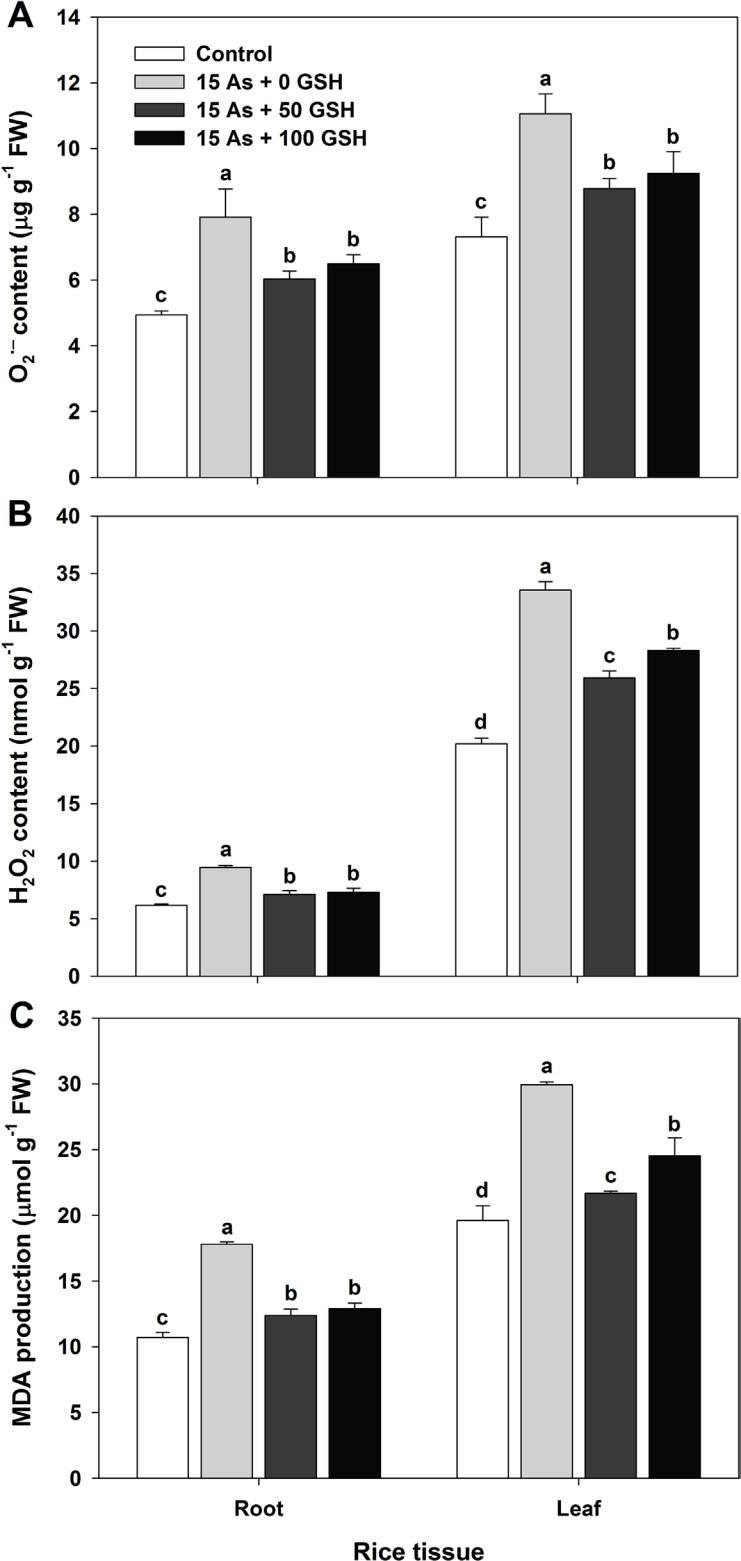
Effect of glutathione (GSH) application on the content of superoxide (**A**; O_2_^•−^), hydrogen peroxide (**B**; H_2_O_2_), and malondialdehyde (**C**; MDA) of rice seedlings grown in the arsenic (As)-treated hydroponics. Rice seedlings at the four-leaf stage were cultivated in a hydroponic solution containing 15 µM of As and sprayed with GSH (50 and 100 mg kg^−1^). Fourteen days after treatment, the content of O_2_^•−^, H_2_O_2_, and MDA in the roots and leaves was measured. The data are presented as mean ± standard error of the mean of three replications. Means denoted by the same letter are not significantly different at the 5% level, according to Fisher’s least significant difference (LSD) test.

The ROS production of the As-treated seedlings was measured on day 14 after GSH application, along with As treatment. The untreated plants were not significantly different on the ROS responses of rice seedlings, compared with the seedlings treated with GSH (data not shown). The O_2_^•−^ content of As-treated plants in the roots and leaves was 1.60- and 1.51-fold higher than that in the untreated seedlings, respectively. However, the O_2_^•−^ content was significantly lower in both roots and leaves of the GSH-treated seedlings, compared with that of the As-treated plants (**Figure 2A**). The H_2_O_2_ content revealed a tendency similar to that of O_2_^•−^ content. The H_2_O_2_ content increased by 154% in the roots and by 166% in the leaves of As-treated seedlings, compared with that of the untreated control. However, the seedlings treated with GSH had significantly lower H_2_O_2_ content of roots and leaves, compared with the As-treated plants (**Figure 2B**). In proportion to the content of O_2_^•−^ and H_2_O_2_, the content of the final product of lipid peroxidation, MDA, was 1.66- and 1.53-fold higher in the roots and leaves of As-treated seedlings than in the controls, respectively. Nonetheless, GSH application significantly reduced the MDA content generated by As toxicity in both the roots and leaves (**Figure 2C**).

### Changes in the Antioxidant Enzymes in As-Stressed Rice Seedlings With or Without GSH Application

**Figure 3:**
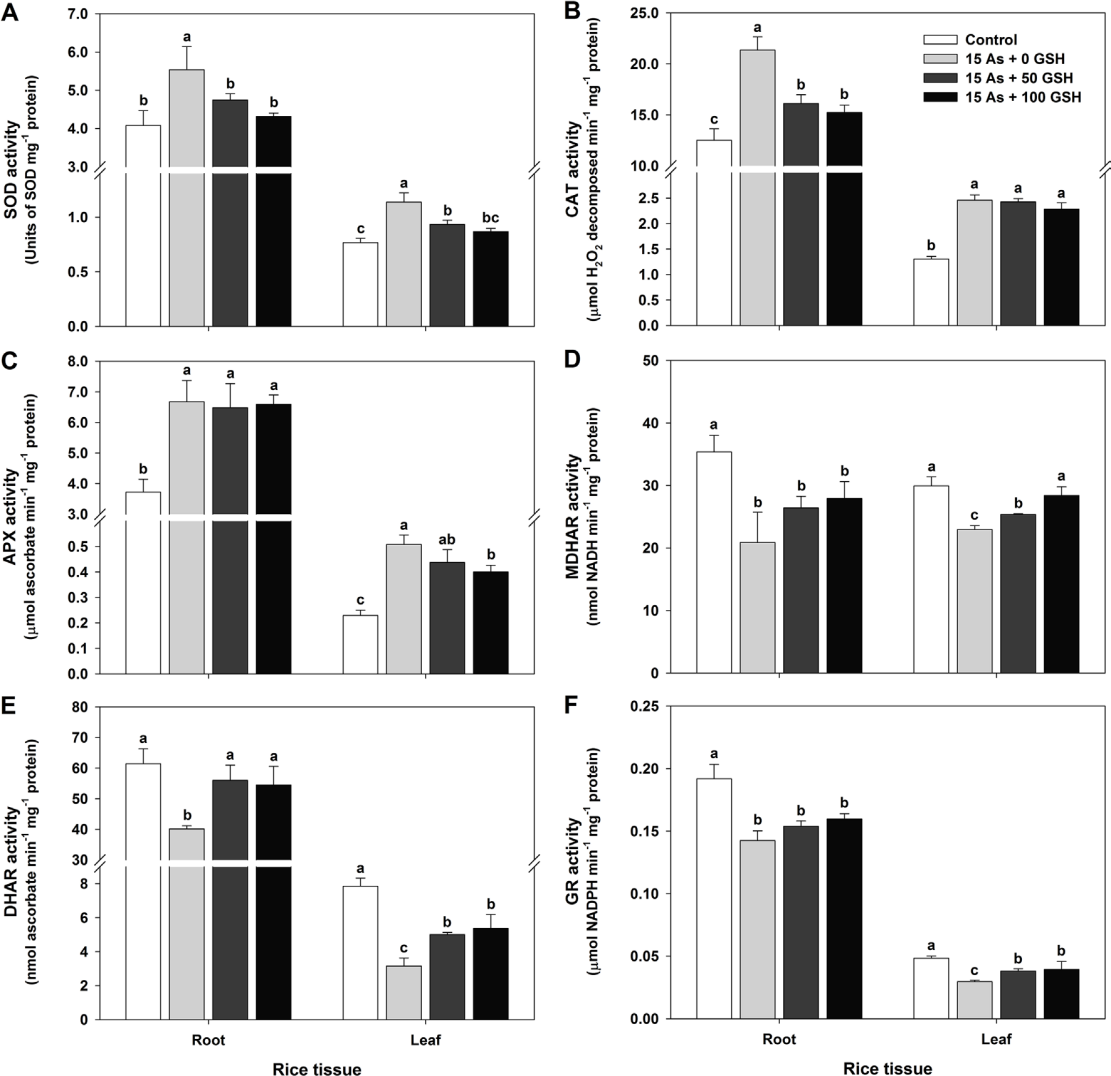
Effect of glutathione (GSH) application on the activity of antioxidant enzymes of rice seedlings grown in arsenic (As)-treated hydroponics; superoxide dismutase (**A**; SOD), catalase (**B**; CAT), ascorbate peroxidase (**C**; APX), monodehydroascorbate reductase (**D**; MDHAR), dehydroascorbate reductase (**E**; DHAR), and glutathione reductase (**F**; GR) activity. Rice seedlings at the four-leaf stage were cultivated in a hydroponic solution containing 15 µM of As and sprayed with GSH (50 and 100 mg kg^−1^). Fourteen days after treatment, the activity of each antioxidant enzymes in the roots and leaves was measured. The data are presented as mean ± standard error of the mean of three replications. Means denoted by the same letter are not significantly different at the 5% level, according to Fisher’s least significant difference (LSD) test.

Since alleviation of the As-induced ROS has been shown in applications of the 50 and 100 mg kg^−1^ GSH to As-stressed plants, the activities of SOD, CAT, APX, MDHAR, DHAR, and GR were evaluated in the roots and leaves to verify the effect of GSH in the As-stressed plants on ROS detoxification. SOD, CAT, and APX activities in the plants treated with As alone were significantly increased in both the roots and leaves (**Figures 3A–C**), whereas MDHAR, DHAR, and GR activities decreased by 41%, 35%, and 26% in the roots and by 23%, 60%, and 39% in the leaves (**Figures 3D–F**), respectively, compared to the untreated plants. Exogenous applications of the 50 and 100 mg kg^−1^ GSH to As-treated seedlings significantly lowered activities of SOD in all organs, compared with plants treated with As alone (**Figure 3A**). The activity of CAT was decreased in the roots but remained unchanged in the leaves of As-treated plants with GSH, compared with As treatment (**Figure 3B**). APX activity in the leaves was reduced but not significantly different in the roots, compared with the As-treated plants (**Figure 3C**). The MDHAR activity in the 50 and 100 mg kg^−1^ GSH-treated seedlings was increased by 126% and 134% in the roots and by 111% and 124% in the leaves, respectively, compared with the As-treated plants (**Figure 3D**). The DHAR and GR activities of GSH-treated plants were significantly higher in both the roots and leaves than those in the As alone treatment (**Figures 3E, F**).

### Changes in the Content of AsA/GSH and DHA/GSSG and the AsA/GSH Redox Ratio of As-Stressed Rice Seedlings With or Without GSH Application

**Figure 4:**
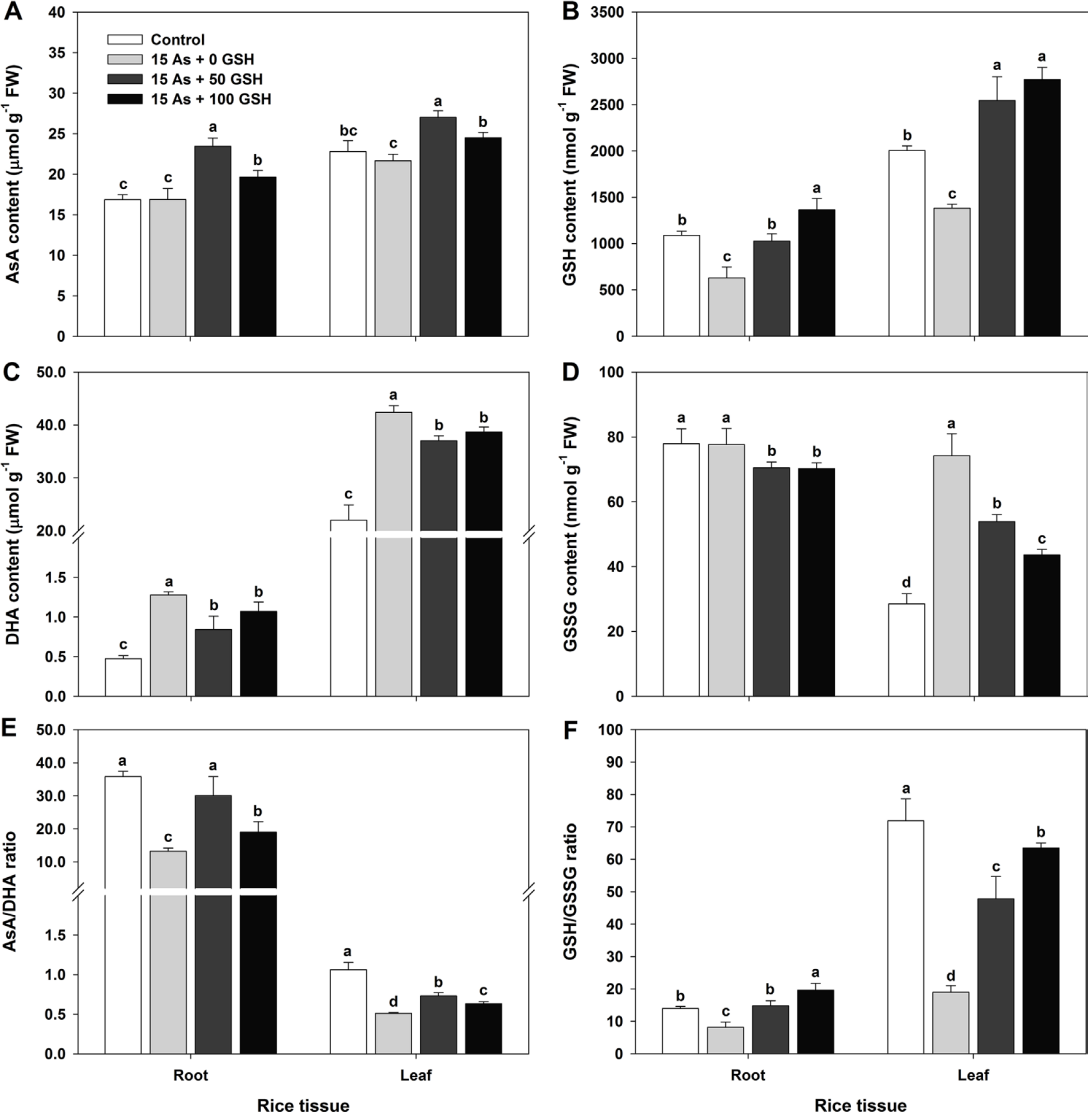
Effect of glutathione (GSH) application on the content of ascorbate (AsA)/glutathione (GSH) and dehydroascorbate (DHA)/GSH disulfide (GSSG) and the AsA/GSH redox ratio in rice seedlings grown in the arsenic (As)-treated hydroponics. Rice seedlings at the four-leaf stage were cultivated in a hydroponic solution containing 15 µM of As and sprayed with GSH (50 and 100 mg kg^−1^). Fourteen days after treatment, the level of AsA/GSH and DHA/GSSG and the AsA/GSH redox ratio in the roots and leaves were measured. **(A** and **B)** Content of AsA and GSH; **(C** and **D)** content of DHA and GSSG; **(E** and **F)** AsA and GSH redox ratios (AsA/DHA and GSH/GSSG). The data are presented as mean ± standard errors of the mean of three replications. Means denoted by the same letter are not significantly different at the 5% level, according to Fisher’s least significant difference (LSD) test.

In plants subjected to As toxicity, the change in redox ratio is closely associated not only with the GSH redox ratio (GSH/GSSG) but also with the AsA redox ratio (AsA/DHA). The rice seedlings exposed to As stress were treated with GSH (50 and 100 mg kg^−1^). The AsA and GSH levels and redox state were assessed on day 14 after GSH treatment along with As application. The AsA level in the As-treated seedlings showed no significant difference in either the roots or leaves, compared with that in the untreated control (**Figure 4A**). However, the DHA level, the oxidized form of AsA, showed significant increases of 270% in the roots and 193% in the leaves, compared with that in As-alone-treated seedlings (**Figure 4C**). The AsA redox ratio decreased significantly by 63% in the roots and by 52% in the leaves, compared with that in the control (**Figure 4E**). The AsA level in the 50 and 100 mg kg^−1^ GSH-treated seedlings was significantly increased in both the roots and leaves, compared with that in As-treated plants (**Figure 4A**). In contrast to the AsA level, the DHA level exhibited a contrary pattern: the DHA level decreased by 34% and 16% in the roots and by 13% and 9% in the leaves (**Figure 4C**). The GSH treatment increased the AsA levels and decreased the DHA levels, compared with As treatment (**Figures 4A, C**). Moreover, GSH treatment significantly increased the AsA redox ratio in both the roots and leaves (**Figure 4E**).

The GSH content and GSH redox ratio of As-treated plants were significantly lower in both the roots and leaves, compared with those in the untreated seedlings (**Figures 4B, F**). The level of GSSG, the oxidized form of GSH, was considerably higher in the leaves and exhibited no change in the roots (**Figure 4D**). The GSH content in the GSH-treated seedlings was dependent on the GSH level (**Figure 4B**). The GSH content was increased in both the roots and leaves (**Figure 4B**), but the GSSG content was decreased in both organs (**Figure 4D**). A high GSH redox ratio was maintained because the GSH treatment increased the GSH content and decreased the GSSG content, compared with that in the As-treated seedlings (**Figures 4B, D, F**).

### Heatmap Responses of Pearson’s Correlation Coefficient (*r*) for the Targeted Antioxidant Metabolites of Oxidative Stress and AsA/GSH Redox State From As-Stressed Rice Seedlings

**Figure 5:**
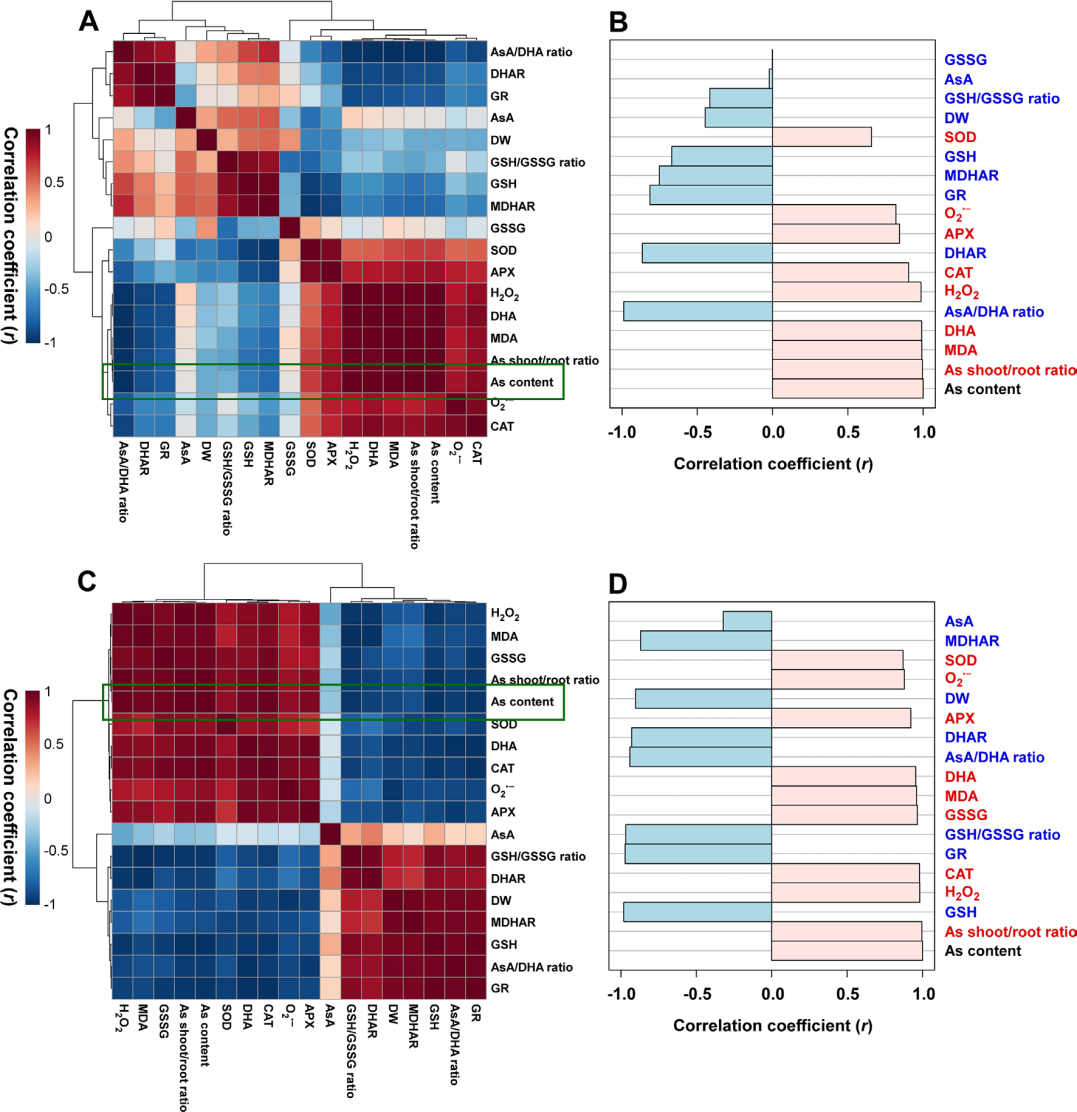
Heatmap responses of Pearson’s correlation coefficient (*r*) for the targeted antioxidant metabolites of oxidative stress and ascorbate (AsA)/glutathione (GSH) redox state in the roots **(A** and **B)** and shoots **(C** and **D)** of the arsenic (As)-stressed seedlings. Red and blue indicate positive and negative correlation coefficients between the targeted antioxidant metabolites. **(B** and **D)** The factors correlated with As content in the roots and shoots, respectively.

With respect to the As-stressed state of the seedlings, the data of heatmap analysis of the roots and leaves were classified into two groups, and each group showed positive correlations. First, a positive correlation was observed between biomass (DW) and redox-associated components. Second, a positive correlation was detected between As content and ROS-related constituents (**Figures 5A, C**). A comparative analysis of the factors related to As content (presented by green boxes) suggested that the roots have a positive correlation with the As shoot/root ratio; MDA, DHA, H_2_O_2_, and O_2_ contents; and CAT, APX, and SOD activities and a negative correlation with the contents of GSH; AsA and GSH redox ratios; DHAR, GR, and MDHAR activities; and DW (**Figure 5B**). On the other hand, the shoots had a positive correlation with the As shoot/root ratio; the contents of H_2_O_2_, MDA, O_2_^•−^, GSSG, and DHA; and CAT, APX, and SOD activities and a negative correlation with the contents of GSH and AsA, DW, GSH and AsA redox ratios, and DHAR, GR, and MDHAR activities (**Figure 5D**).

### PCA of the Targeted Antioxidant Metabolites of Oxidative Stress and AsA/GSH Redox State From Rice Seedlings

**Figure 6:**
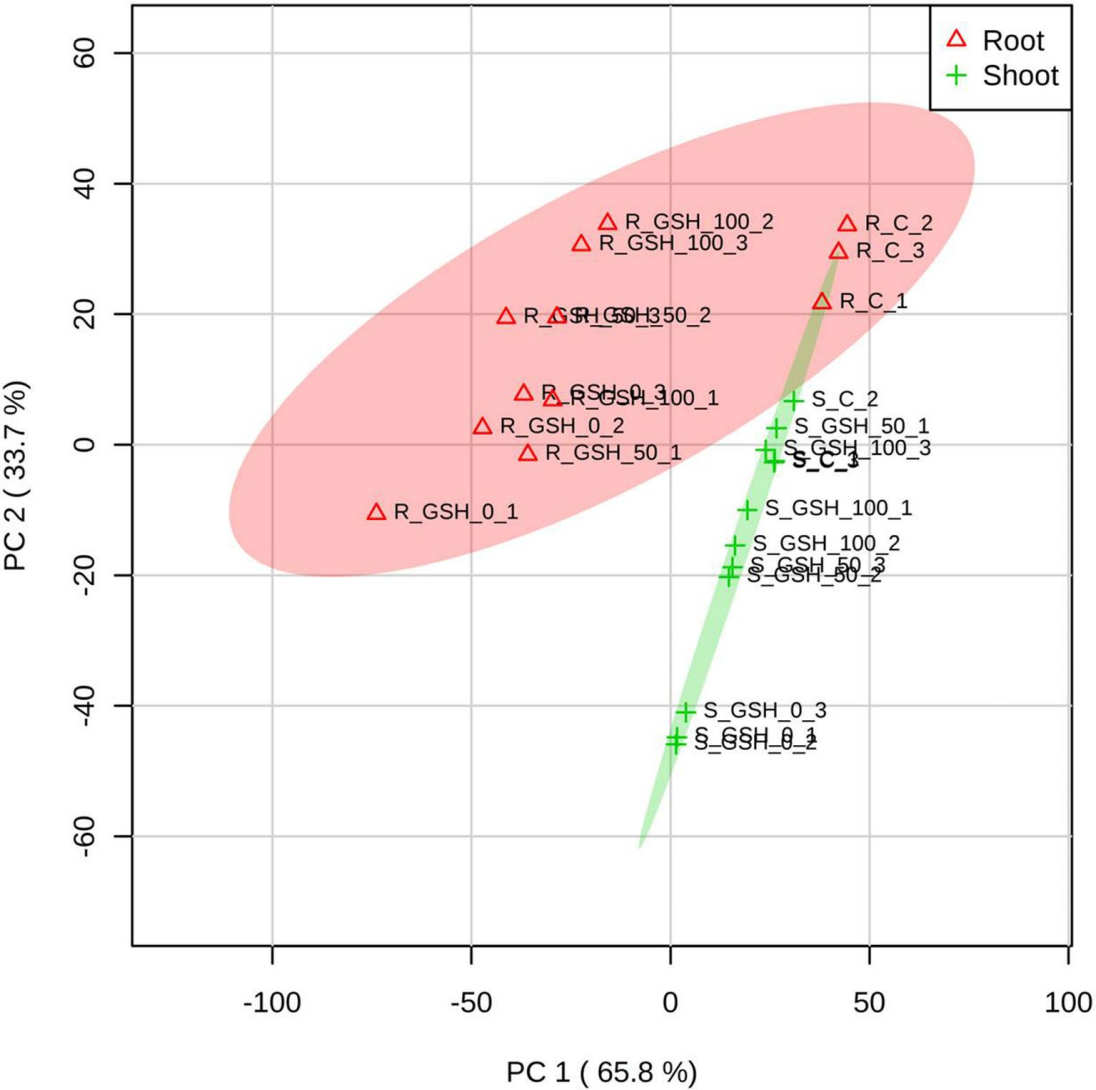
Principal component analysis (PCA) score plots of the targeted antioxidant metabolites of oxidative stress and ascorbate (AsA)/glutathione (GSH) redox state in the roots (∆) and shoots (+) of seedlings treated with different GSH levels. R: root, S: shoot. The numbers after R or S of the code names indicate the GSH level (0, 50, or 100 mg GSH kg^−1^). The last digit indicates which of the three replicates is shown.

The PCA score plot was applied to evaluate the overall responses of rice seedlings to GSH treatment under As stress conditions. The score plot accounted for 65.8% and 33.7% of the variance of principal components 1 and 2, respectively. The analysis results indicated that both the tissues (root and shoot) exhibited a similar pattern of plant physiological responses according to the GSH levels. The responses of the two tissues became similar to those of the untreated with an increase in the GSH level. These results suggest that As stress alleviation is GSH concentration dependent. In addition, the effect of GSH treatment was more evident in the shoots than in the roots (**Figure 6**).

## Discussion

The pollution of agricultural soils and waters by As contamination has led to serious environmental problems in the productivity of crop plants. As toxicity affects photosynthesis by reducing the chlorophyll content, which negatively influences general plant growth and important metabolic processes, and by inhibiting adenosine triphosphate (ATP) biosynthesis by disrupting phosphorus uptake in the roots (Panda et al., 2010; Finnegan and Chen, 2012; Gupta and Ahmad, 2014; Jung et al., 2017b). Recently, some studies have demonstrated that the exogenous application of hydrogen sulfide (Singh et al., 2015b; Li et al., 2016; Chen et al., 2017), sulfur (Khan et al., 2015; Jung et al., 2017a; Tian et al., 2017), AsA (Jung et al., 2018; Semida et al., 2018), or GSH (Shri et al., 2009; Chen et al., 2010; Mostofa et al., 2014; Kim et al., 2017) may alleviate different heavy metal stresses.

In this study, As-stressed rice seedlings showed overall growth inhibition caused by As toxicity, regardless of GSH treatments (**Table 1**). However, significantly lower As concentrations were observed in the roots of As-exposed rice seedlings treated with GSH compared with those treated with As alone. In contrast, the shoot As content of plants treated with both As and GSH was significantly higher than that of plants treated with As alone (**Figure 1A**). Studies have reported that As uptake and translocation differ by plant species and tissues (Ye et al., 2012; Gupta and Ahmad, 2014; Chen et al., 2015; Jung et al., 2017b). The results of this study revealed that GSH application mitigates the overall phytotoxicity of As by decreasing the content of As within the roots but increasing its translocation to the shoots (**Figure 1**).

The uptake of As promotes the production of ROS, O_2_^•−^, H_2_O_2_, and OH^•^ and thus oxidative stress, which is considered to be primarily responsible for oxidative toxicity in plant cells. Furthermore, it disturbs metabolic pathways and redox homeostasis (Mostofa et al., 2014; Farooq et al., 2016). These complex biochemical effects can cause lipid peroxidation, which ultimately withers the rice plant *via* the destruction of cell membranes (Jung et al., 2017a; Sharma et al., 2017). This study demonstrated that As induced oxidative stresses by the overproduction of O_2_^•−^ (**Figure 2A**) and H_2_O_2_ (**Figure 2B**) in the roots and leaves of rice seedlings. This is in agreement with the findings of Shri et al. (2009), Singh et al. (2015a) and Singh et al. (2016). However, GSH application to As-stressed rice seedlings decreased the level of O_2_^•−^ and H_2_O_2_ and thus played a critical role in alleviating the oxidative stress caused by As. Increased levels of ROS lead to lipid peroxidation causing membrane damage and malfunctioning of membrane proteins and ion transporters (Sharma, 2012; Jung et al., 2015; Rahman et al., 2015). MDA serves as an indicator of lipid peroxidation under oxidative stress conditions. In association with ROS, the MDA production was increased significantly (**Figure 2C**), which indicated severe membrane damage in the As-stressed seedlings. However, As-induced production of O_2_^•−^, H_2_O_2_, and MDA decreased significantly with the exogenous application of GSH to the As-stressed seedlings (**Figure 2**). The GSH-mediated decrease in oxidative stress is *via* its antioxidant effect in the direct or indirect ROS scavenging systems, thus decreasing MDA production, restricting As uptake, or altering the antioxidant scavenging system involved in decreasing ROS levels.

**Figure 7:**
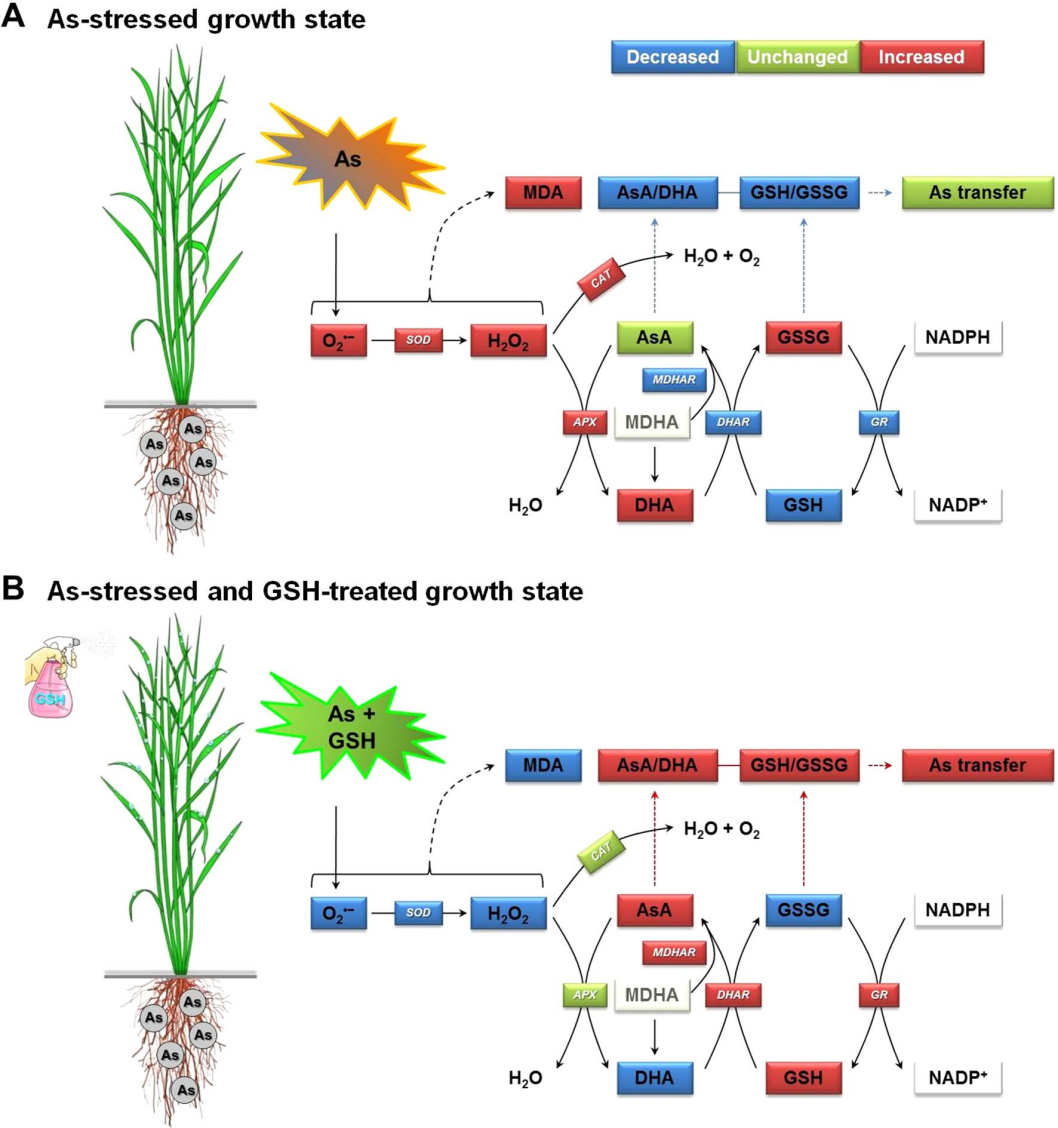
Proposed mechanism of increase in As transfer in rice seedlings by alleviating As-induced oxidative stress and maintaining homeostasis of the AsA–GSH cycle, which includes two interdependent redox couples, AsA/DHA and GSH/GSSG. **(A)** The As-stressed plant growth state and **(B)** the As-exposed and GSH-treated plant growth state. APX, ascorbate peroxidase; AsA, ascorbate (reduced form); As, arsenic; DHA, dehydroascorbate (oxidized form); AsA/DHA, AsA/DHA redox ratio; CAT, catalase; DHAR, dehydroascorbate reductase; GR, glutathione reductase; GSH, glutathione (reduced form); GSSG, glutathione disulfide (oxidized form); GSH/GSSG, GSH/GSSG redox ratio; MDA, malondialdehyde (an index chemical of lipid peroxidation); MDHA, monodehydroascorbate; MDHAR, monodehydroascorbate reductase; NADPH (reduced form) and NADP^+^ (oxidized form), nicotinamide adenine dinucleotide phosphate; SOD, superoxide dismutase.

The detoxification strategies of As-induced oxidative stress alleviate As toxicity by inducing enzymatic and non-enzymatic antioxidant scavenging systems. These systems play a critical role in protecting the structure and function of membrane systems and in sustaining cellular redox states (Shri et al., 2009; Chen et al., 2010; Foyer and Noctor, 2011; Farooq et al., 2016; Jung et al., 2017a; Sharma et al., 2017). In the enzymatic antioxidant or redox enzymes, SOD plays a critical role as it catalyzes the dismutation of O_2_^•−^ to H_2_O_2_. Subsequently, CAT and APX convert H_2_O_2_ to H_2_O and O_2_ for scavenging of the oxidative damages (Farooq et al., 2016; Hayyan et al., 2016; Akram et al., 2017; Sharma et al., 2017). In the present study, high levels of SOD, CAT, and APX activities under As-stressed condition were observed in the plants, while their activities reduced significantly with the application of GSH to the As-stressed rice plants (**Figures 2** and **3**). The amending effects of GSH application could be correlated with decreased ROS levels in the As-stressed plants, thereby minimizing the risk of oxidative stress (**Figure 2**). In contrast, the core enzymes, which were involved in the AsA/GSH redox cycle and MDHAR, DHAR, and GR activities in the roots and leaves of rice plants, were markedly decreased under As stress. However, the exogenous application of GSH significantly increased the activities of MDHAR, DHAR, and GR in rice plants, except for the MDHAR and GR activities of the roots, compared with the As-alone treatment (Shri et al., 2009) (**Figure 3**). Results described that application of GSH improved the protective ability of plants by changing antioxidant enzymatic activities and also depressed the levels of O_2_^•−^, H_2_O_2_, and MDA in rice plants, thereby providing further resistance to the plants against oxidative stress produced by As. In addition, alterations of antioxidant enzymes under As stress were reduced by the application of GSH with the activities recovering to close to those in the untreated control plants. On the other hand, another antioxidant mechanism is a non-enzymatic system that uses antioxidative or redox molecules, including GSH, to protect against As-induced ROS damages and sustain normal redox balances in plant cells (Foyer and Noctor, 2011). GSH is a key component in the AsA–GSH cycle, and it is a precursor of phytochelatins (PCs), which are mainly involved in heavy metal detoxification by sequestrating them in the vacuoles (Noctor et al., 2012; Mostofa et al., 2014; Kim et al., 2017; Sharma et al., 2017; Semida et al., 2018). However, the exogenous application of GSH increases the biosynthesis of PCs, which subsequently chelate As and alter its translocation. Furthermore, an altered As speciation upon GSH treatment could also affect translocation (Shri et al., 2009). In this study, As treatment decreased the GSH levels in all organs of rice plants, but not significantly different in the AsA levels (**Figures 4A, B**). Interestingly, there was an increase in the content of DHA and GSSG (**Figures 4C, D**), whereas the AsA/DHA and GSH/GSSG ratios were concomitantly decreased (**Figures 4E, F**). In **Figures 2** **–** **6**, the results demonstrate that the oxidative stress induced by As in rice plants should be associated with the redox status of AsA (AsA, DHA, and AsA/DHA ratio) and GSH (GSH, GSSG, and GSH/GSSG ratio). However, GSH application increased the AsA and GSH levels and AsA/DHA and GSH/GSSG ratios, which could strengthen the inductions of the redox cycle to suppress ROS caused by As toxicity. Consistent with our results, Shri et al. (2009) and Mostofa et al. (2014) reported that exogenous GSH treatment alleviated As- and Cu-induced ROS, lowered lipid peroxidation, increased GSH levels, and helped maintain the cellular redox homeostasis in rice seedlings. In summary, GSH application to As-stressed rice seedlings significantly decreased the production of O_2_^•−^, H_2_O_2_, and MDA, relative to that observed in plants subjected to As treatment alone. However, the application of GSH to rice plants under As stress stimulated the AsA–GSH cycle inductions, thereby promoting ROS scavenging, which decreased overall As toxicity (**Figure 7**). The AsA–GSH cycle plays an important role in sustaining the balance between ROS production and scavenging in plants that are exposed to many abiotic stresses, including drought, high temperature, and heavy metal stresses. The altered induction of the AsA–GSH cycle observed under As or Cd stress is shown to be a result of the need to sustain a favorable redox status. This is achieved by conserving appropriate levels of AsA and GSH to overcome the potential problems of oxidation (Ranieri et al., 1996; Foyer and Noctor, 2011; Jung et al., 2017a).

## Conclusions

GSH is an antioxidant in plants, which is capable of preventing damage to important cellular components caused by ROS, such as O_2_^•−^ and H_2_O_2_. Our study provided an understanding into the role of GSH in regulating the physiological and biochemical responses to As stress. This study demonstrated that As stress caused oxidative stress, which was evidenced by the high levels of O_2_^•−^, H_2_O_2_, and MDA associated with the insufficient or inhibitive induction of the enzymatic and non-enzymatic antioxidant systems. However, the exogenous GSH application to rice seedlings under As stress significantly enhanced antioxidant enzymes’ activities and non-enzymatic antioxidants, which finally reduced As-induced oxidative stresses. In addition, exogenous GSH enhanced the induction of the AsA–GSH cycle, which re-established the cellular redox status. Therefore, the results suggest that application with exogenous GSH should be a potential approach to enhance As stress resistance in rice plants.

## Author Contributions

H-iJ and Y-HK designed the experiment and wrote the manuscript. H-iJ, M-SK, and B-RL performed the experiment. H-iJ, Y-HK, M-SK, B-RL, T-HK, E-JL, G-BJ, M-JC, C-HL, and J-KS analyzed the data and provided constructive comments and comprehensive discussion on the manuscript.

## Conflict of Interest Statement

The authors declare that the study was conducted in the absence of any commercial or financial relationships that could be construed as a potential conflict of interest.

## References

[B1] Akinbile C. O. Haque A. M. M. (2012). Arsenic contamination in irrigation water for rice production in Bangladesh: a review. Trends Appl. Sci. Res. 7, 331–349. 10.3923/tasr.2012.331.349

[B2] Akram N. A. Shafiq F. Ashraf M. (2017). Ascorbicacid—a potential oxidant scavenger and its role in plant development and abiotic stress tolerance. Front. Plant Sci. 8, 613. 10.3389/fpls.2017.00613 28491070 PMC5405147

[B3] Buege J. A. Aust S. D. (1978). Microsomal lipid peroxidation. Methods Enzymol. 52, 302–310. 10.1016/S0076-6879(78)52032-6 672633

[B4] Chen F. Wang F. Wu F. Mao W. Zhang G. Zhou M. (2010). Modulation of exogenousglutathione in antioxidant defense system against Cd stress in the two barley genotypes differing in Cd tolerance. Plant Physiol. Biochem. 48, 663–672. 10.1016/j.plaphy.2010.05.001 20605723

[B5] Chen G. X. Asada K. (1989). Ascorbate peroxidase in tea leaves: occurrence of two isozymes and the differences in their enzymatic and molecular properties. Plant Cell Physiol. 30, 987–998. 10.1093/oxfordjournals.pcp.a077844

[B6] Chen Y. Moore K. L. Miller A. J. McGrath S. P. Ma J. F. Zhao F. J. (2015). The role of nodes in arsenic storage and distribution in rice. J. Exp. Bot. 66, 3717–3724. 10.1093/jxb/erv164 25922485 PMC4473974

[B7] Chen Z. Chen M. Jiang M. (2017). Hydrogen sulfide alleviates mercury toxicity by sequestering it in roots or regulating reactive oxygen species productions in rice seedlings. Plant Physiol. Biochem. 111, 179–192. 10.1016/j.plaphy.2016.11.027 27940269

[B8] Dave R. Tripathi R. D. Dwivedi S. Tripathi P. Dixit G. Sharma Y. K. (2013). Arsenate and arsenite exposure modulate antioxidants and amino acids in contrasting arsenic accumulating rice (*Oryza sativa* L.) genotypes. J. Hazard. Mater. 15, 1123–1131. 10.1016/j.jhazmat.2012.06.049 22917495

[B9] Elstner E. F. Heupel A. (1976). Inhibition of nitrite formation from hydroxylammonium-chloride: a simple assay for superoxide dismutase. Anal. Biochem. 70, 616–620. 10.1016/0003-2697(76)90488-7 817618

[B10] Farooq M. A. Gill R. A. Islam F. Ali B. Liu H. Xu J. (2016). Methyl jasmonate regulates antioxidant defense and suppresses arsenicuptake in *Brassica napus* L. Front. Plant Sci. 7, 468. 10.3389/fpls.2016.00468 27148299 PMC4826882

[B11] Finnegan P. M. Chen W. (2012). Arsenictoxicity: the effects on plantmetabolism. Front. Physiol. 3, 182. 10.3389/fphys.2012.00182 22685440 PMC3368394

[B12] Foyer C. H. Noctor G. (2011). Ascorbate and glutathione: the heart of the redox hub. Plant Physiol. 155, 2–18. 10.1104/pp.110.167569 21205630 PMC3075780

[B13] Gupta M. Ahmad M. A. (2014). Arsenate induced differential response in rice genotypes. Ecotoxicol. Environ. Saf. 107, 46–54. 10.1016/j.ecoenv.2014.04.030 24905696

[B14] Hayyan M. Hashim M. A. AlNashef I. M. (2016). Superoxide ion: generation and chemical implications. Chem. Rev. 116, 3029–3085. 10.1021/acs.chemrev.5b00407 26875845

[B15] Hossain M. A. Nakano Y. Asada K. (1984). Monodehydroascorbate reductase in spinach chloroplasts and its participation in the regeneration of ascorbate for scavenging hydrogen peroxide. Plant Cell Physiol. 25, 385–395. 10.1093/oxfordjournals.pcp.a076726

[B16] Jana S. Choudhuri M. A. (1982). Glycolate metabolism of three submerged aquatic angiosperms during ageing. Aquat. Bot. 12, 345–354. 10.1016/0304-3770(82)90026-2

[B17] Jung H. I. Chae M. J. Kim S. J. Kong M. S. Kang S. S. Lee D. B. (2015). Effects of cadmium and arsenic on physiological responses and copper and zinc homeostasis of rice. Kor. J. Soil Sci. Fert. 48, 397–403. 10.7745/KJSSF.2015.48.5.397

[B18] Jung H. I. Kong M. S. Chae M. J. Lee E. J. Jung G. B. Kim Y. H. (2018). Effect of ascorbate on the arsenic uptake, ROS-scavenging capacity, and antioxidant homeostasis in rice. Kor. J. Soil Sci. Fert. 51, 90–100. 10.7745/KJSSF.2018.51.2.090

[B19] Jung H. I. Lee B. R. Chae M. J. Kong M. S. Lee C. H. Kang S. S. (2017a). Sulfur alleviates cadmium toxicity in rice (*Oryza sativa* L.) seedlings by altering antioxidant levels. J. Crop Sci. Biotech. 20, 213–220. 10.1007/s12892-017-0072-0

[B20] Jung H. I. Lee J. Chae M. J. Kong M. S. Lee C. H. Kang S. S. (2017b). Growth-inhibition patterns and transfer-factor profiles in arsenic-stressed rice (*Oryza sativa* L.). Environ. Monit. Assess. 189, 638. 10.1007/s10661-017-6350-3 29147882 PMC5691118

[B21] Kamachi K. Yamaya T. Mae T. Ojima K. (1991). A role for glutamine synthetase in the remobilization of leaf nitrogen during natural senescence in rice leaves. Plant Physiol. 96, 411–417. 10.1104/pp.96.2.411 16668201 PMC1080785

[B22] Khan M. A. Stroud J. L. Zhu Y. G. McGrath S. P. Zhao F. J. (2010). Arsenic bioavailability to rice is elevated in Bangladeshi paddy soils. Environ. Sci. Technol. 44, 8515–8521. 10.1021/es101952f 20977268

[B23] Khan M. I. Nazir F. Asgher M. Per T. S. Khan N. A. (2015). Selenium and sulfurinfluenceethyleneformation and alleviate cadmium-induced oxidative stress by improving proline and glutathione production in wheat. J. Plant Physiol. 173, 9–18. 10.1016/j.jplph.2014.09.011 25462073

[B24] Kim H. S. Kang D. W. Kim D. I. Lee S. Park S. W. Yoo J. H. (2016). Inorganic As concentration in rice grown around the abandoned mining areas and its health risk assessment. Kor. J. Soil Sci. Fert. 49, 584–588. 10.7745/KJSSF.2016.49.5.584

[B25] Kim Y. O. Bae H. J. Cho E. Kang H. (2017). Exogenous glutathioneenhances mercury tolerance by inhibiting mercury entry into plantcells. Front. Plant Sci. 8, 683. 10.3389/fpls.2017.00683 28507557 PMC5410599

[B26] Law M. Y. Charles S. A. Halliwell B. (1983). Glutathione and ascorbic acid in spinach (*Spinacia oleracea*) chloroplasts: the effect of hydrogen peroxide and of paraquat. Biochem. J. 210, 899–903. 10.1042/bj2100899 6307273 PMC1154305

[B27] Lee B. R. Li L. S. Jung W. J. Jin Y. L. Avice J. C. Qurry A. (2009). Water deficit-induced oxidative stress and the activation of antioxidant enzymes in white clover leaves. Biol. Plant. 53, 505–510. 10.1007/s10535-009-0091-2

[B28] Lee B. R. Muneer S. Park S. H. Zhang Q. Kim T. H. (2013). Ammonium-induced proline and sucrose accumulation, and their significance in antioxidative activity and osmotic adjustment. Acta Physiol. Plant. 35, 2655–2664. 10.1007/s11738-013-1297-7

[B29] Li Z. G. Min X. Zhou Z. H. (2016). Hydrogen sulfide: asignal molecule in plantcross-adaptation. Front. Plant Sci. 7, 1621. 10.3389/fpls.2016.01621 27833636 PMC5080339

[B30] Meharg A. A. Hartley-Whitaker J. (2002). Arsenic uptake and metabolism in arsenic resistant and nonresistant plant species. New Phytol. 154, 29–43. 10.1046/j.1469-8137.2002.00363.x

[B31] Meister A. Anderson M. E. (1983). Glutathione. Annu. Rev. Biochem. 52, 711–760. 10.1146/annurev.bi.52.070183.003431 6137189

[B32] Mostofa M. G. Seraj Z. I. Fujita M. (2014). Exogenoussodiumnitroprusside and glutathionealleviatecoppertoxicity by reducing copper uptake and oxidative damage in rice (*Oryza sativa* L.) seedlings. Protoplasma 251, 1373–1386. 10.1007/s00709-014-0639-7 24752795

[B33] Nakano Y. Asada K. (1981). Hydrogen peroxide is scavenged by ascorbate-specific peroxidase in spinach chloroplasts. Plant Cell Physiol. 22, 867–880. 10.1093/oxfordjournals.pcp.a076232

[B34] Noctor G. Mhamdi A. Chaouch S. Han Y. Neukermans J. Marquez-Garcia B. (2012). Glutathione in plants: an integratedoverview. Plant Cell Environ. 35, 454–484. 10.1111/j.1365-3040.2011.02400.x 21777251

[B35] Panda S. K. Upadhyay R. K. Nath S. (2010). Arsenic stress in plants. J. Agron. Crop Sci. 196, 161–174. 10.1111/j.1439-037X.2009.00407.x

[B36] Petrov V. Hille J. Mueller-Roeber B. Gechev T. S. (2015). ROS-mediated abiotic stress-induced programmed cell death in plants. Front. Plant Sci. 6, 69. 10.3389/fpls.2015.00069 25741354 PMC4332301

[B37] Rahman A. Mostofa M. G. Alam M. M. Nahar K. Hasanuzzaman M. Fujita M. (2015). Calcium mitigates arsenictoxicity in riceseedlings by reducing arsenic uptake and modulating the antioxidant defense and glyoxalase systems and stress markers. Biomed. Res. Int. 2015, 340812. 10.1155/2015/340812 26798635 PMC4698539

[B38] Ranieri A. D’Urso G. Nali C. Lorenzini G. Soldatini G. G. (1996). Ozone stimulates apoplastic antioxidant systems in pumpkin leaves. Physiol. Plant. 97, 381–387. 10.1034/j.1399-3054.1996.970224.x

[B39] Rao M. V. Paliyath G. Ormrod D. P. (1996). Ultraviolet B- and ozone-induced biochemical changes in antioxidant enzymes of *Arabidopsis thaliana*. Plant Physiol. 110, 125–136. 10.1104/pp.110.1.125 8587977 PMC157701

[B40] Roychowdhury T. Uchino T. Tokunaga H. Ando M. (2002). Survey of arsenic in food composites from an arsenic-affected area of West Bengal, India. Food Chem. Toxicol. 40, 1611–1621. 10.1016/S0278-6915(02)00104-7 12176088

[B41] Semida W. M. Hemida K. A. Rady M. M. (2018). Sequenced ascorbate–proline–glutathione seed treatment elevates cadmium tolerance in cucumber transplants. Ecotoxicol. Environ. Saf. 154, 171–179. 10.1016/j.ecoenv.2018.02.036 29471279

[B42] Sharma I. (2012). Arsenic induced oxidative stress in plants. Biologia 67, 447–453. 10.2478/s11756-012-0024-y

[B43] Sharma S. Anand G. Singh N. Kapoor R. (2017). Arbuscular mycorrhiza augments arsenictolerance in wheat (*Triticum aestivum* L.) by strengthening antioxidant defense system and thiol metabolism. Front. Plant Sci. 8, 906. 10.3389/fpls.2017.00906 28642762 PMC5462957

[B44] Shri M. Kumar S. Chakrabarty D. Trivedi P. K. Mallick S. Misra P. (2009). Effect of arsenic on growth, oxidative stress, and antioxidant system in rice seedlings. Ecotoxicol. Environ. Saf. 72, 1102–1110. 10.1016/j.ecoenv.2008.09.022 19013643

[B45] Singh A. P. Dixit G. Kumar A. Mishra S. Singh P. K. Dwivedi S. (2016). Nitric oxide alleviated arsenictoxicity by modulation of antioxidants and thiol metabolism in rice (*Oryza sativa* L.). Front. Plant Sci. 6, 1272. 10.3389/fpls.2015.01272 26793232 PMC4709823

[B46] Singh A. P. Dixit G. Mishra S. Dwivedi S. Tiwari M. Mallick S. (2015a). Salicylic acid modulates arsenic toxicity by reducing its root to shoot translocation in rice (*Oryza sativa* L.). Front. Plant Sci. 6, 340. 10.3389/fpls.2015.00340 26042132 PMC4434920

[B47] Singh V. P. Singh S. Kumar J. Prasad S. M. (2015b). Hydrogen sulfide alleviates toxic effects of arsenate in pea seedlings through up-regulation of the ascorbate–glutathione cycle: possible involvement of nitric oxide. J. Plant Physiol. 181, 20–29. 10.1016/j.jplph.2015.03.015 25974366

[B48] Singh V. P. Srivastava P. K. Prasad S. M. (2013). Nitric oxide alleviates arsenic-induced toxic effects in ridged *Luffa* seedlings. Plant Physiol. Biochem. 71, 155–163. 10.1016/j.plaphy.2013.07.003 23917073

[B49] Tian M. Hui M. Thannhauser T. W. Pan S. Li L. (2017). Selenium-induced toxicity is counteracted by sulfur in broccoli (*Brassica oleracea* L. *var. italica*). Front. Plant Sci. 8, 1425. 10.3389/fpls.2017.01425 28868057 PMC5563375

[B50] Williams P. N. Price A. H. Raab A. Hossain S. A. Feldmann J. Meharg A. A. (2005). Variation in arsenic speciation and concentration in paddy rice related to dietary exposure. Environ. Sci. Technol. 39, 5531–5540. 10.1021/es0502324 16124284

[B51] Xia J. Wishart D. S. (2016). Using MetaboAnalyst 3.0 for comprehensive metabolomics data analysis. Curr. Protoc. Bioinf. 55, 14.10.1–14.10.91. 10.1002/cpbi.11 27603023

[B52] Xie Z. M. Huang C. Y. (1998). Control of arsenic toxicity in rice plants grown on an arsenic-polluted paddy soil. Commun. Soil Sci. Plant Anal. 29, 2471–2477. 10.1080/00103629809370125

[B53] Ye X. X. Sun B. Yin Y. L. (2012). Variation of As concentration between soiltypes and ricegenotypes and the selection of cultivars for reducing As in the diet. Chemosphere 87, 384–389. 10.1016/j.chemosphere.2011.12.028 22221666

[B54] Zhu Y. G. Williams P. N. Meharg A. A. (2008). Exposure to inorganic arsenic from rice: a global health issue? Environ. Pollut. 154, 169–171 10.1016/j.envpol.2008.03.015 18448219

